# Preparation of new diatomite–chitosan composite materials and their adsorption properties and mechanism of Hg(II)

**DOI:** 10.1098/rsos.170829

**Published:** 2017-12-13

**Authors:** Yong Fu, Xiaoxu Xu, Yue Huang, Jianshe Hu, Qifan Chen, Yaoqing Wu

**Affiliations:** 1 Center for Molecular Science and Engineering, College of Science, Northeastern University, Shenyang 110819, People's Republic of China; 2 School of Chemical Engineering, Eastern Liaoning University, Dandong 118001, People's Republic of China

**Keywords:** chitosan, diatomite, absorbent, absorption kinetic model, mercury ions

## Abstract

A new composite absorbent with multifunctional and environmental-friendly structures was prepared using chitosan, diatomite and polyvinyl alcohol as the raw materials, and glutaraldehyde as a cross-linking agent. The structure and morphology of the composite absorbent, and its adsorption properties of Hg(II) in water were characterized with Fourier transform infrared (FT-IR) spectra, scanning electron microscope (SEM), X-ray diffraction (XRD), Brunauer Emmett Teller (BET) measurements and ultraviolet–visible (UV–Vis) spectra. The effect of the pH value and contact time on the removal rate and absorbance of Hg(II) was discussed. The adsorption kinetic model and static adsorption isotherm and regeneration of the obtained composite absorbent were investigated. The results indicated that the removal of Hg(II) on the composite absorbent followed a rapid adsorption for 50 min, and was close to the adsorption saturation after 1 h, which is in accord with the Langmuir adsorption isotherm model and the pseudo-second-order kinetic model. When the pH value, contact time and the mass of the composite absorbent was 3, 1 h and 100 mg, respectively, the removal rate of Hg(II) on the composite absorbent reached 77%, and the maximum adsorption capacity of Hg(II) reached 195.7 mg g^−1^.

## 1. Introduction

As is known, water is one of the basic necessities required for the sustenance and continuation of life. It is, therefore, important that good quality water should be available for various activities. However, this is becoming increasingly difficult in view of large-scale pollution caused by industrial, agricultural and personal activities. These activities generate wastewater which contains many heavy metals. Although some heavy metals such as copper, selenium and zinc are essential to human life and health, they become harmful to living species when taken in excess [1]. With regard to heavy metal pollution, mercury ions are the most prominent in water pollution [2,3]. Mercury pollution is largely caused by volcanic and geothermal activity, and the deposition of atmospheric mercury has been an important source of mercury in soil and water. In recent years, wastewaters from the plastic industry, chlorine-alkali industry and electronic industry are the main sources of mercury ion pollution in water. Therefore, how to remove the mercury ions in wastewater has attracted more and more attention.

Many types of methods have been studied for the treatment of aqueous stream contaminated with heavy metal ions, such as chemical precipitation [4–6], electro-dialysis [7], adsorption methods, and so on. Among them, the adsorption method has become the most effective, economic and accessible method. In recent years, different types of adsorbents such as polymers [8–10], amorphous silica [11–14] and clays [15–19] have been reported. However, these adsorption materials have some shortcomings, such as low mechanical and thermal stability, poor removal efficiency and high cost, therefore it is very important to develop new adsorption materials.

Today, material science is directed towards the development of multifunctional and environmental-friendly structures. As a natural macromolecule material, chitosan has drawn particular attention as a potential effective sorbent due to its low cost and high content of active functional groups such as amino and hydroxyl groups. In this context, various chitosan–natural or synthetic polymers composites, such as natural and cross-linked chitosan [20], chemical modification of chitosan [21], chitosan/cellulose [22], chitosan/polyacrylamide [23] and chitosan/phenylthiourea resin [24], have been applied to adsorption of various heavy metals. However, they are expensive materials, with complicated processes or poor mechanical stability, which limits their application for industrial applications. Immobilizing chitosan on cheap materials, therefore, is necessary to improve the mechanical stability of adsorbents. Chitosan-coated clays, such as chitosan/bentonite [25], chitosan/montmorillonite [26], chitosan/perlite [27] and chitosan/sand [28], have been reported to remove various heavy metals from water, and the participation of cheap clays reduces the amount of chitosan needed to synthesize the composite material and improves mechanical stability of the composite material. As a clay mineral, diatomite has large surface area, a great number of channels, many active groups and negative charge. Diatomite is a cheap and environmental mineral material, and has a large adsorptive capacity and no secondary pollution. However, to the best of our knowledge, little research on composite adsorption materials based on diatomite and chitosan using glutaraldehyde as a cross-linking agent has been reported.

In this work, we designed and prepared a new environmentally-friendly composite absorbent based on diatomite and chitosan. Compared with the conventional composite adsorbents (CAs), this composite absorbent not only had high removal rate and adsorption capacity, but also improved the mechanical stability of the adsorbent and reduced cost. The structure and morphology of the obtained composite absorbent were characterized with Fourier transform infrared (FT-IR) spectra, scanning electron microscope (SEM) and X-ray diffraction (XRD) measurements. Its adsorption properties of Hg(II) in water was investigated by ultraviolet–visible (UV–Vis) spectra. The static non-equilibrium adsorption isotherm and static equilibrium adsorption isotherm were discussed.

## 2. Experimental set-up

### 2.1. Material

The raw diatomite (92.8% SiO_2_, 4.2% Al_2_O_3_, 1.5% Fe_2_O_3_ and other metal oxides) was purchased from Jilin Kaida Diatomite Co. Ltd. (Jilin, China). Chitosan (CS, 85% deacetylated) was purchased from Qingdao Baicheng Biochemical Co. (Qingdao, China). Glutaraldehyde (25%) was purchased from Damao Chemical Agent Company (Tijin, China). Mercurium nitrate was purchased from Guizhou Tongren Tailuier chemical plant (Guizhou, China).

### 2.2. Characterization

FT-IR spectra were measured on a PerkinElmer Spectrum One (B) spectrometer (PerkinElmer, Foster City, CA, USA). The morphology was observed by scanning electron microscope (SEM, JEOL 6500F, Japan). X-ray diffraction (XRD) measurements were performed using a DMAX-3A Rigaku XRD powder diffractometer (Bruker, Germany) with a nickel-filtered Cu-Kα radiation at a scan rate of 0.02 s^−1^ in 2*θ* range (10°–80°). The Brunauer–Emmett–Teller (BET) surface area and the pore size distribution were measured using N_2_ adsorption and desorption (Quadrasorb SI, Quantachrome, USA) at 77 K. The mercury ion concentration was measured by a TU-1901 dual-beam UV–Vis spectrophotometer (Beijing Purkinje General Instrument Co., Ltd., China) with a range of 450–600 nm.

### 2.3. Preparation of new composite adsorbent

The diatomite was treated with 40% sulfuric acid at a solid–liquid ratio of 1 : 4, after which the mixture was stirred at 80°C for 8 h, filtered, washed several times with distilled water and dried at 60°C for 24 h. The dried diatomite was placed in a muffle furnace and calcined at 450°C for 6 h, and then sealed for storage.

In a 1000 ml three-neck flask, a mixture of 350 ml of 2% chitosan acetic acid solution, 27 ml of 10% polyvinyl alcohol solution and 3 g purified diatomite was stirred vigorously. After the mixture was maintained for 4 h at room temperature, 7.5 ml of 1% glutaraldehyde was added dropwise to the mixture and continuously stirred for 4 h. The crude product was placed in a freezer for 24 h at −18°C, and then 12 h at room temperature. The process was repeated three times. Finally, the product was placed in a diluted sodium hydroxide solution for 24 h and washed to neutral with deionized water. The cross-linking diatomite/chitosan powder CA was obtained by drying until a constant sample mass was obtained.

### 2.4. Adsorption experiments

The adsorption experiments were performed by using CA as a new adsorbent. First, the pH value of mercury nitrate solution was adjusted by adding a small amount of 0.1 M HCl or NaOH solution. Secondly, a certain amount of adsorbent was added to the mercury nitrate solution and adsorption equilibrium was obtained through stirring. Finally, the concentration of Hg(II) in water was detected with dithizone spectrophotometry.

## 3. Results and discussion

### 3.1. Structural characterization

**Figure 1.**
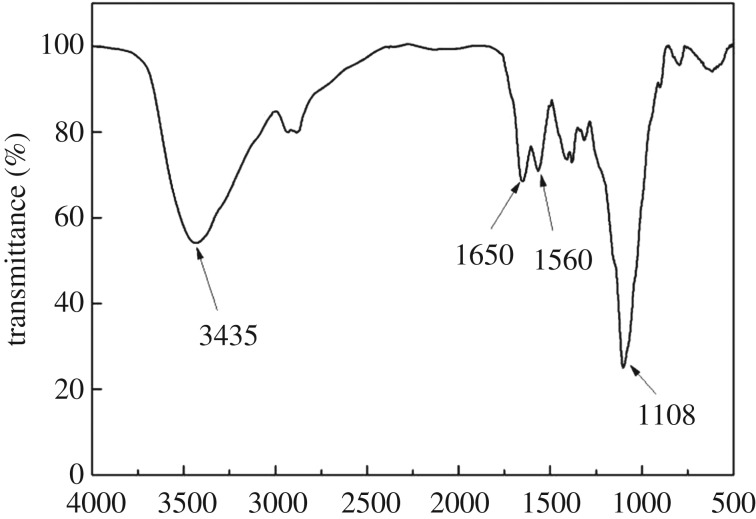
FT-IR spectrum of CA.

The structure of the obtained CA was characterized by FT-IR, SEM, XRD and Brunauer Emmett Teller (BET) measurement. The FT-IR spectrum of CA, shown in figure 1, exhibited characteristic bands at 3435, 1650 and 1108 cm^−1^, attributed to ─OH, ─NH_2_, acid amides and Si─O stretching vibration peaks, respectively. In addition, the bank appearing at 1560 cm^−1^ belonged to a C═N stretching vibration peak, which showed the formation of a Schiff base in CA due to the cross-linking reaction of glutaraldehyde with amino groups of chitosan.

**Figure 2.**
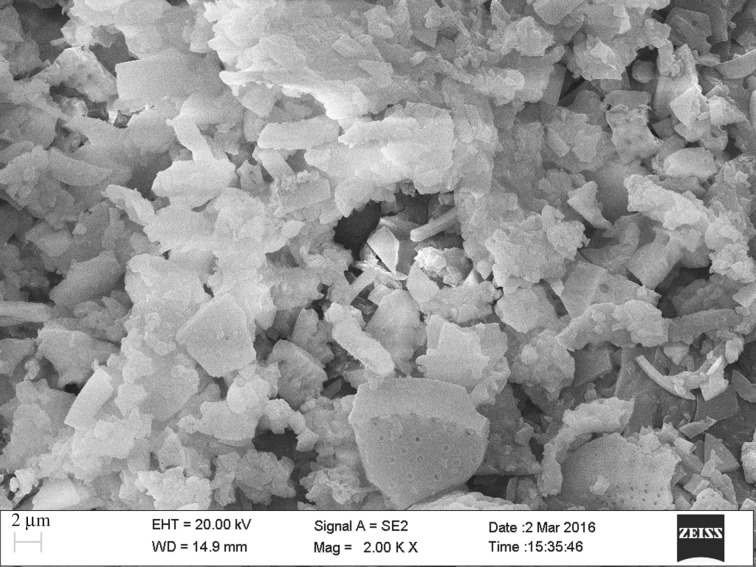
SEM image of CA.

**Figure 3.**
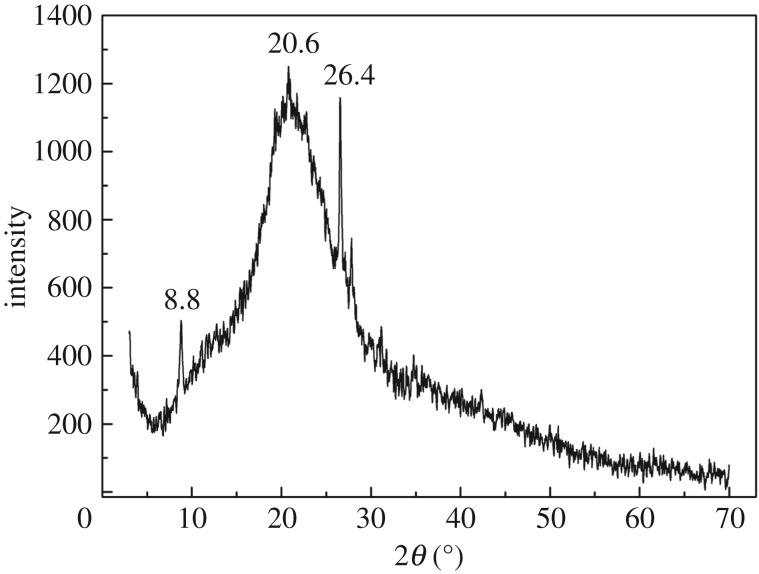
XRD pattern of CA.

The SEM image of CA is shown in figure 2. As can been clearly seen, the particles of chitosan were loaded on the surface of the diatomite particles. To further identify the structure of CA, XRD studies were carried out. Figure 3 shows the XRD patterns of CA powder at room temperature. Two peaks at 2*θ* of 20.6° and 26.4° belonged to typical SiO_2_ diffraction peaks, and a characteristic peak at 8.8° was attributed to a chitosan diffraction peak. This is the result of the formation of strong molecular and intermolecular hydrogen bonding between ─OH and ─NH_2_ in chitosan molecules; this cross-linking reaction makes the structure of the diatomaceous soil layer relatively stable. Specific surface area and pore structure of CA and diatomite were measured and shown in table 1. Immobilizing chitosan on diatomite blocked the channels of some of the diatomite, resulting in reduction of the pore volume and pore size of CA. However, the specific surface area of CA was larger than diatomite because chitosan was cross-linked with glutaraldehyde to form some new micropores.

**Table 1.** Surface area, pore diameter and pore volume of CA and diatomite.

sample	pore volume (cm^3^ g^−1^)	pore size (nm)	surface area (m^2^ g^−1^)
diatomite	0.12	14.95	7.70
CA	0.11	4.21	15.04

### 3.2. Adsorption properties

**Figure 4.**
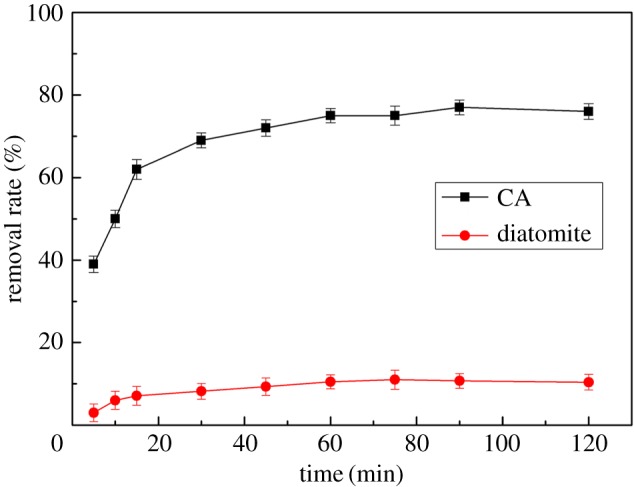
Effect of CA and diatomite on the adsorption of Hg(II).

To investigate the effect of CA on the adsorption properties of Hg(II), 100 ml of standard solution of mercury ions (200 mg l^−1^) was added to two small beakers with 100 mg CA and 100 mg diatomite for 1 h at 20°C, respectively. Figure 4 shows the effect of CA and diatomite on the adsorption of Hg(II). The adsorptive capacity of CA for Hg(II) was more than that of the diatomite. So the efficiency of modified diatomite/chitosan CA was very high.

**Figure 5.**
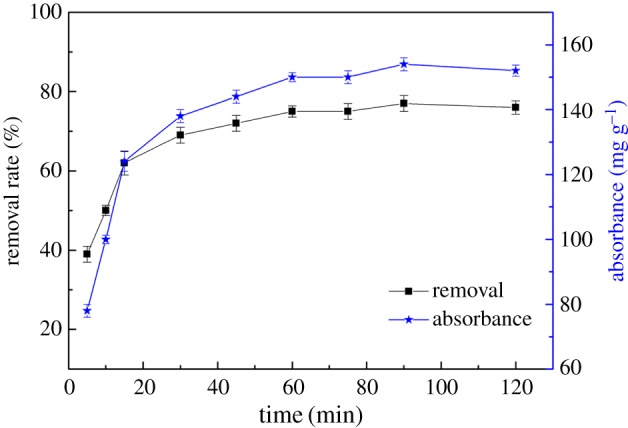
Effect of contact time on the adsorption of Hg(II).

Figure 5 shows the effect of contact time on the adsorption of Hg(II). According to figure 5, the adsorptive capacity of CA for Hg(II) increased with time. In the initial stage of adsorption, the removal rate of Hg(II) rapidly increased when the contact time was less than 45 min because ─NH_2_ and ─OH groups in chitosan and micropores of the diatomite surface had a double adsorption effect on Hg(II). However, after 45 min the adsorption effect slowed down, and after 60 min the removal rate showed almost no change. The results were explained as follows: as adsorption proceeded and the surface of CA tended to saturate, mercury ions could further spread inside the adsorbent, but because of the existence of diffusion resistance, the removal rate by CA of Hg(II) was obviously less than the initial surface adsorption rate.

**Figure 6.**
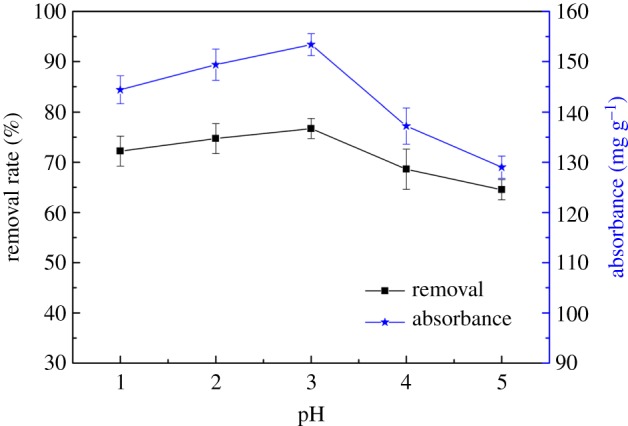
Effect of pH on the adsorption of Hg(II).

Figure 6 shows the effect of pH on the adsorption of Hg(II). It can be seen from figure 6 that CA had a relative good adsorption capacity for Hg(II) when the pH value of the solution was in the range from 1 to 5. The maximum removal rate of CA achieved 77% when the pH value was 3. When the pH value was more than 7, white flocculation appeared due to the generation of Hg(OH)_2_ precipitation.

### 3.3. Regeneration of adsorbent

**Figure 7.**
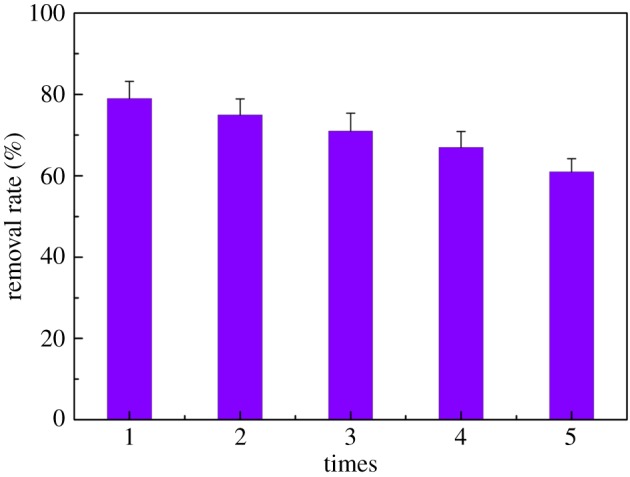
Reuse effect of CA for the adsorption of Hg(II).

To confirm a reversible process and re-use of CA, an experiment was performed as follows. First, 100 mg CA was placed in 200 mg l^−1^ of mercury nitrate solution, adsorbed and then separated. Second, this CA was added to 50 ml of saturated EDTA solution as a desorbent, and the mixture was stirred for 3 h. Finally, the concentration of mercury ions was detected by dithizone spectrophotometry. Figure 7 shows the reuse effect of CA.

After performing the cycle five times, the removal rate of mercury ions decreased from 79% to 62%. CA acts as an adsorbent at pH 3; in this condition, a small amount of chitosan is dissolved in solution, resulting in the loss of associated adsorbent material during the repeated cycles, which is one of the main reasons for the decrease of recyclability. The results illuminate that the adsorbents could be effectively regenerated by HNO_3_, indicating that CA had a better reusable performance.

### 3.4. Kinetic model analysis

In order to describe the adsorption mechanism and the speed steps of the adsorption process, the pseudo-first-order kinetic model and the pseudo-second-order kinetic model were adopted based on the experimental data.

The equation of the pseudo-first-order kinetic model was as follows:

3.1 $$\ln(q_{e}-q_{t})=\ln q_{e}-k_{1}t$$

where *t* is contact time, *q*_e_ (mg g^−1^) and *q_t_* (mg g^−1^) are the adsorption capacity at equilibrium and at contact time *t* and *k*_1_ (min^−1^) represents the pseudo-order reaction rate constant.

The equation of the pseudo-second-order kinetic model was described as follows:

3.2 $$\frac{t}{q_{e}}=\frac{1}{k_{2}{q_{e}}^{2}}+\frac{1}{q_{e}t}$$

where *k*_2_ (g mg^−1^ min^−1^) is the pseudo-second-order reaction rate constant.

**Figure 8.**
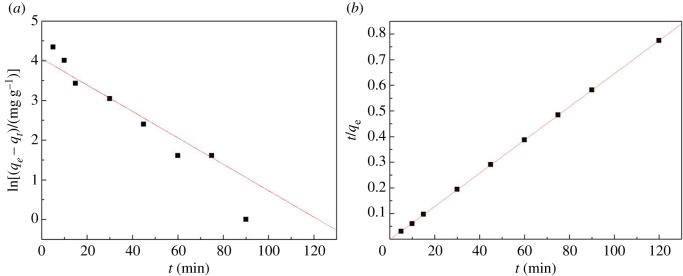
Kinetic model CA for the adsorption of Hg(II). (*a*) First-order kinetic model and (*b*) second-order kinetic model.

Figure 8 shows the fitting results of the first- and second-order kinetic models. It could be seen that the adsorption of mercury ions to CA was in agreement with the pseudo-second-order kinetics model.

As shown in table 2, the adsorption equilibrium capacity value of the first-order kinetic model was 58.32 mg g^−1^, while the experimental adsorption balance capacity value was 155 mg g^−1^. However, for the second-order kinetic model, the capacity value of adsorption equilibrium was 160.5 mg g^−1^, and it had only a difference of 5.5 mg g^−1^ from the balance capacity value. Thus, this indicated that the adsorption of mercury ions to CA was well in agreement with the second-order kinetic model.

**Table 2.** Pesudo-first- and second-order kinetic parameters for the adsorption of Hg(II).

	pseudo-first-order kinetics			pseudo-second-order kinetics		
*q*_e-exp_^a^	*q*_e-cal_^b^	*K*_1_	*R*^2^	*q*_e-cal_^b^	*K*_2_	*R*^2^
155	58.32	0.0358	0.8647	160.5	0.1664	0.9991

^a^Data obtained by experiment.

^b^Data obtained by calculation.

### 3.5. Adsorption isotherms

The isothermal adsorption equation for metal ions is described by the Langmuir model and Freundlich model. Both can be used for physical and chemical adsorption. Their adsorption isotherm equations is described as follows:

3.3 $$\frac{C_{e}}{q_{e}}=\frac{1}{K_{L}q_{m}}+\frac{C_{e}}{q_{m}}$$

and

3.4 $$\mathrm{lg}q_{e}=\mathrm{lg}K_{F}+\frac{1}{n}\mathrm{lg}C_{e}$$

where *C*_e_ (mg l^−1^) is the equilibrium concentration for heavy metal ions, *q*_e_ (mg g^−1^) is the adsorption capacity, *q*_m_ (mg g^−1^) is the maximum adsorption capacity, *K*_F_ (l mg^−1^) and *K*_L_ (l mg^−1^) represent the Freundlich and Langmuir adsorption constant, respectively, and *n* is the adsorption intensity.

**Figure 9.**
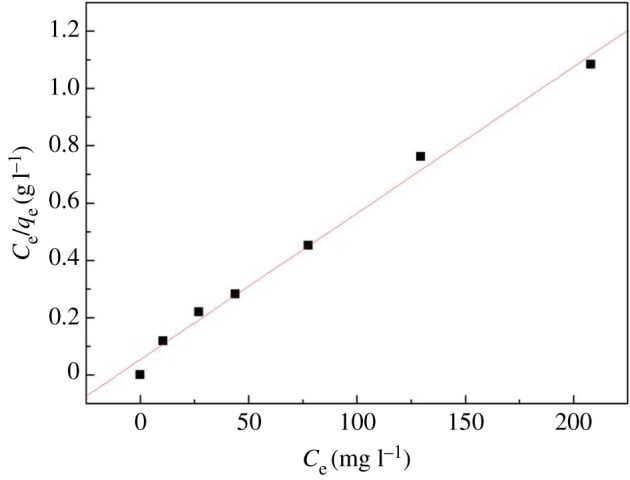
Langmuir adsorption isotherms.

**Figure 10.**
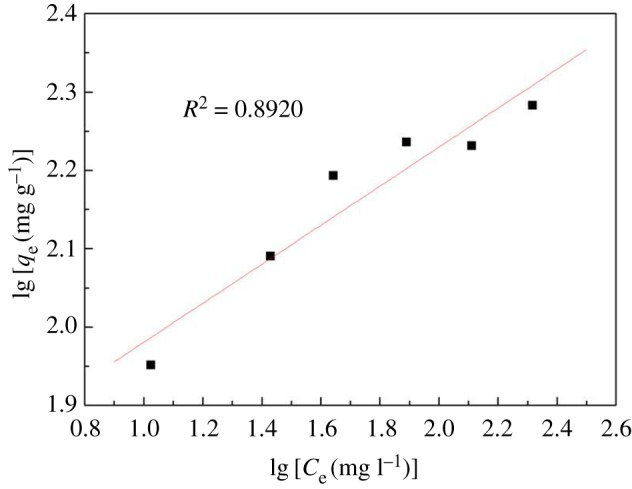
Freundlich adsorption isotherms.

Figures 9 and 10 show linear regression for the Langmuir equation *C*_e_/*q*_e−_*C*_e_ and Freundlich equation lg*q*_e_ − lg*C*_e_. According to Freundlich adsorption, *K*_F_ and 1/*n* were obtained by the intercept and slope of the straight line of lg*q*_e_ versus lg*C*_e_, respectively. The corresponding Langmuir and Freundlich parameters of CA for the adsorption of Hg(II) are shown in table 3.

**Table 3.** Langmuir and Freundlich parameters of CA for the adsorption of Hg(II).

Langmuir			Freundlich		
*q*_m_	*K*_L_	*R*^2^	*n*	*K*_F_	*R*^2^
195.7	0.09466	0.9906	4.014	53.83	0.8920

Comparing figures 9 and 10, the Langmuir adsorption isotherm can better describe the adsorption behaviour of CA towards Hg(II). The CA obtained in this study for the adsorption of Hg(II) was in the form of a monolayer adsorption.

As shown in table 3, CA was more applicable to the Langmuir model, with the maximum adsorption capacity of CA for Hg(II) of 195.7 mg g^−1^.

### 3.6. Comparison with other studies

Table 4 compares the best adsorption capacity of CA with different CS composite sorbents for the removal of Hg(II) ions. It can be clearly seen in the table that the adsorption capacity of CA is higher than that of most adsorbents. In short, considering the process characteristics of this work, CA as a low-cost sorbent not only decreases the amount of chitosan needed using cheap diatomite but also has high adsorption capacity and good mechanical stability, which indicates that the CA will be a good candidate for applications in heavy metal removal from wastewater.

**Table 4.** Maximum adsorption capacity (*q*_max_) values of Hg(II) ions on CA compared with other reported adsorbents in the literature.

adsorbent	*q*_max_ (mg g^−1^)	references
chitosan–polyacrylamide	89	[23]
chitosan–phenylthiourea resin	135	[24]
chitosan–poly(vinyl alcohol) hydrogel	586	[29]
chitosan–cotton fibres	104	[30]
chitosan–diatomite	196	this work

## 4. Conclusion

In this study, a new composite absorbent was prepared by a cross-linking reaction of chitosan, diatomite and glutaraldehyde. When the pH-value, contact time and mass of the composite absorbent was 3, 1 h, and 100 mg, respectively, the removal rate of Hg(II) on the composite absorbent reached 77%. The results of the static non-equilibrium adsorption isotherm showed that the removal of Hg(II) on the composite absorbent followed a rapid adsorption for 50 min, and was close to adsorption saturation after 1 h, which followed the pseudo-second-order kinetic model. The results of the static equilibrium adsorption isotherm showed that the adsorption of Hg(II) was according to the Langmuir adsorption isotherm model, and the maximum adsorption capacity of Hg(II) reached 195.7 mg g^−1^ at the same conditions. In addition, it was found that the adsorption capacity of Hg(II) decreased little after four times re-use of the composite absorbent, which indicated that this composite absorbent had good regeneration behaviour.

## Data accessibility

This paper has no additional data.

## References

[1] Yang Y, Wu WQ, Zhou HH, Huang Z-Y, Ye T, Liu R, Kuang Y-f 2014 Adsorption behavior of cross-linked chitosan modified by graphene oxide for Cu(II) removal. J. Cent. South Univ. 21, 2826–2831. (doi:10.1007/s11771-014-2246-3)

[2] Tenório JAS, Espinosa DCR 2001 Treatment of chromium plating process effluents with ion exchange resins. Waste Manag. 21, 637–642. (doi:10.1016/S0956-053X(00)00118-5) 11530919 10.1016/s0956-053x(00)00118-5

[3] Cheng HF, Hu YN 2012 Mercury in municipal solid waste in China and its control: a review. Environ. Sci. Technol. 46, 593–605. (doi:10.1021/es2026517) 22136661 10.1021/es2026517

[4] Khraisheh MA, Aldegs YS, Mcminn WA 2004 Remediation of wastewater containing heavy metals using raw and modified diatomite. Chem. Eng. J. 99, 177–184. (doi:10.1016/j.cej.2003.11.029)

[5] Charerntanyarak L 1999 Heavy metals removal by chemical coagulation and precipitation. Water Sci. Technol. 39, 135–138.

[6] Chen QY, Luo Z, Hills C, Xue G, Tyrer M 2009 Precipitation of heavy metals from wastewater using simulated flue gas: sequent additions of fly ash, lime and carbon dioxide. Water Res. 43, 2605–2614. (doi:10.1016/j.watres.2009.03.007) 19375144 10.1016/j.watres.2009.03.007

[7] Amor Z, Bariou B, Mameri N, Taky M, Nicolas S, Elmidaoui A 2001 Fluoride removal from brackish water by electrodialysis. Desalination 133, 215–223. (doi:10.1016/S0011-9164(01)00102-3)

[8] Weismantel M, Funk R, De Kaey R 2013 Method for manufacturing water-absorbing polymer particles with a low centrifuge retention capacity. Patent No. US 8497336 B2.

[9] Craeye B, Geirnaert M, Schutter GD 2011 Super absorbing polymers as an internal curing agent for mitigation of early-age cracking of high-performance concrete bridge decks. Const. Build. Mater. 25, 1–13. (doi:10.1016/j.conbuildmat.2010.06.063)

[10] Rogers JH 2012 Plasma confinement rings including RF absorbing material for reducing polymer deposition. Patent No. US8337662.

[11] Liu D, Yuan P, Tan D, Liu H, Fan M, Yuan A, Zhu J, He H 2010 Effects of inherent/enhanced solid acidity and morphology of diatomite templates on the synthesis and porosity of hierarchically porous carbon. Langmuir 26, 18624–18627. (doi:10.1021/la103980s) 21080632 10.1021/la103980s

[12] Zhu J, Wang P, Wu X 2012 Adsorption of Pb^2+^ ions on diatomite modified by polypropylene acetamide and barium chloride in aqueous solution. Span. J. Agric. Res. 24, 3614–3620.

[13] Rasouli M, Yaghobi N, Hafezi M 2012 Adsorption of divalent lead ions from aqueous solution using low silica nano-zeolite X. J. Ind. Eng. Chem. 18, 1970–1976. (doi:10.1016/j.jiec.2012.05.014)

[14] Goswami A, Singh AK 2002 Silica gel functionalized with resacetophenone: synthesis of a new chelating matrix and its application as metal ion collector for their flame atomic absorption spectrometric determination. Anal. Chim. Acta. 454, 229–240. (doi:10.1016/S0003-2670(01)01552-5)

[15] Azad MM, Smith SJ, Joy M 2012 Superabsorbent polymer containing clay, particulate, and method of making same. Patent No. US 8222477 B2.

[16] Islam MS, Rahaman MS, Yeum JH 2015 Electrospun novel super-absorbent based on polysaccharide–polyvinyl alcohol–montmorillonite clay nanocomposites. Carbohyd. Polym. 115, 69–77. (doi:10.1016/j.carbpol.2014.08.086) 10.1016/j.carbpol.2014.08.086 25439870

[17] Patra SK, Swain SK 2011 Swelling study of superabsorbent PAA-co-PAM/clay nanohydrogel. J. Appl. Polym. Sci. 120, 1533–1538. (doi:10.1002/app.33381)

[18] Kalaleh HA, Tally M, Atassi Y 2013 Preparation of a clay based superabsorbent polymer composite of copolymer poly(acrylate-co-acrylamide) with bentonite via microwave radiation. Physics 4, 145–150.

[19] Sarkar S, Datta SC, Biswas DR 2015 Effect of fertilizer loaded nanoclay/superabsorbent polymer composites on nitrogen and phosphorus release in soil. Proc. Natl Acad. Sci. Ind. B 85, 415–421. (doi:10.1007/s40011-014-0371-2)

[20] Vieira RS, Beppu MM 2005 Mercury ion recovery using natural and crosslinked chitosan membranes. Adsorption 11, 731–736. (doi:10.1007/s10450-005-6015-3)

[21] Jeon C, Höll WH 2003 Chemical modification of chitosan and equilibrium study for mercury ion removal. Water Res. 37, 4770–4780. (doi:10.1016/S0043-1354(03)00431-7) 14568064 10.1016/S0043-1354(03)00431-7

[22] Wan Ngah WS, Teong LC, Hanafiah MAKM 2011 Adsorption of dyes and heavy metal ions by chitosan composites: a review. Carbohyd. Poly. 83, 1446–1456. (doi:10.1016/j.carbpol.2010.11.004)

[23] Li K, Wang Y, Huang M, Yan H, Yang H, Xiao S, Li A 2015 Preparation of chitosan-graft-polyacrylamide magnetic composite microspheres for enhanced selective removal of mercury ions from water. J. Colloid Interface Sci. 455, 261–270. (doi:10.1016/j.jcis.2015.05.043) 26073848 10.1016/j.jcis.2015.05.043

[24] Monier M, Abdellatif DA 2012 Preparation of cross-linked magnetic chitosan-phenylthiourea resin for adsorption of Hg(II), Cd(II) and Zn(II) ions from aqueous solutions. J. Hazard. Mater. 209–210, 240–249. (doi:10.1016/j.jhazmat.2012.01.015) 10.1016/j.jhazmat.2012.01.015 22277339

[25] Futalan CM, Kan CC, Dalida MLP, Hsien KJ, Pascua C, Wan MW 2011 Comparative and competitive adsorption of copper, lead, and nickel using chitosan immobilized on bentonite. Carbohydr. Polym. 83, 528–536. (doi:10.1016/j.carbpol.2010.08.013)

[26] Fan DH, Zhu XM, Xu MR, Yan J 2006 Adsorption properties of chromium (VI) by chitosan coated montmorillonite. J. Biol. Sci. 6, 941–945.

[27] Boddu VM, Abburi K, Randolph AJ, Smith ED 2008 Removal of copper (II) and nickel (II) ions from aqueous solutions by a composite chitosan biosorbent. Sep. Sci. Technol. 43, 1365–1381. (doi:10.1080/01496390801940762)

[28] Wan MW, Kan CC, Rogel BD, Dalida MLP 2010 Adsorption of copper (II) and lead (II) ions from aqueous solution on chitosan-coated sand. Carbohydr. Polym. 80, 891–899. (doi:10.1016/j.carbpol.2009.12.048)

[29] Wang X, Deng W, Xie Y, Wang C 2013 Selective removal of mercury ions using a chitosan–poly(vinyl alcohol) hydrogel adsorbent with three-dimensional network structure. Chem. Eng. J. 228, 232–242. (doi:10.1016/j.cej.2013.04.104)

[30] Qu R, Sun C, Ma F, Zhang Y, Ji C, Xu Q, Wang C, Chen H 2009 Removal and recovery of Hg(II) from aqueous solution using chitosan-coated cotton fibers. J. Hazard. Mater. 167, 717–727. (doi:10.1016/j.jhazmat.2009.01.043) 19201531 10.1016/j.jhazmat.2009.01.043

